# Association of Treatment Intensity With Survival in Older Patients With Hodgkin Lymphoma

**DOI:** 10.1001/jamanetworkopen.2021.28373

**Published:** 2021-10-21

**Authors:** Angie Mae Rodday, Theresa Hahn, Anita J. Kumar, Peter K. Lindenauer, Jonathan W. Friedberg, Andrew M. Evens, Susan K. Parsons

**Affiliations:** 1 Institute for Clinical Research and Health Policy Studies, Tufts Medical Center, Boston, Massachusetts; 2 Roswell Park Comprehensive Cancer Center, Buffalo, New York; 3 Institute for Healthcare Delivery and Population Science, University of Massachusetts Medical School Baystate, Springfield; 4 Wilmot Cancer Institute, Rochester, New York; 5 Rutgers Cancer Institute of New Jersey, New Brunswick

## Abstract

**Importance:**

Hodgkin lymphoma is an aggressive blood cancer that is highly curable in younger patients who receive multiagent chemotherapy. Worse survival in older patients may reflect less-aggressive treatment, competing risks of death, or different disease biological factors.

**Objective:**

To examine the association between treatment intensity and cause-specific mortality among older adults with Hodgkin lymphoma.

**Design, Setting, and Participants:**

This was a population-based cohort study of patients aged 65 years or older with Medicare Part A and B fee-for-service coverage who received a diagnosis of Hodgkin lymphoma from 2000 to 2013. The association between treatment intensity and cause-specific mortality was estimated separately for early-stage and advanced-stage disease with Cox proportional hazards models. Multivariable adjustment and propensity score weighting helped control for confounding. Data are from the 1999 to 2016 Surveillance, Epidemiology, and End Results Medicare database. Data analysis was performed from April 2020 to June 2021.

**Exposures:**

First-line treatment categorized as (1) full chemotherapy regimen, (2) partial chemotherapy regimen, (3) single chemotherapy agent or radiotherapy, or (4) no treatment.

**Main Outcomes and Measures:**

The main outcome was 3-year Hodgkin lymphoma–specific and other-cause mortality.

**Results:**

Among 2686 patients (mean [SD] age, 75.7 [6.9] years; 1333 men [50%]), 1307 had early-stage disease and 1379 had advanced-stage disease. For Hodgkin lymphoma–specific mortality in patients with early-stage disease, hazard ratios (HRs) were higher for partial regimens (HR, 1.77; 95% CI, 1.22-2.57) or no treatment (HR, 1.91; 95% CI, 1.31-2.79) than for full regimens; there was no difference between single-agent chemotherapy or radiotherapy and full regimens. For other-cause mortality in patients with early-stage disease, HRs were higher for partial regimens (HR, 1.69; 95% CI, 1.18-2.44), single-agent chemotherapy or radiotherapy (HR, 1.62; 95% CI, 1.13-2.33), or no treatment (HR, 2.71; 95% CI, 1.95-3.78) than for full regimens. For Hodgkin lymphoma–specific mortality in patients with advanced-stage disease, HRs were higher for partial regimens (HR, 3.26; 95% CI, 2.44-4.35), single-agent chemotherapy or radiotherapy (HR, 2.85; 95% CI, 1.98-4.11), or no treatment (HR, 4.06; 95% CI, 3.06-5.37) than for full regimens. For other-cause mortality in patients with advanced-stage disease, HRs were higher for partial regimens (HR, 1.76; 95% CI, 1.32-2.33), single-agent chemotherapy or radiotherapy (HR, 1.65; 95% CI, 1.15-2.37), or no treatment (HR, 2.24; 95% CI, 1.71-2.94) than for full regimens.

**Conclusions and Relevance:**

This cohort study found variability in the magnitude of the association between treatment intensity and mortality by stage and cause-specific mortality, possibly reflecting competing risks of death. However, full chemotherapy regimens were associated with lower mortality and could be considered for older adults who can tolerate them.

## Key Points

**Question:**

Is treatment with full, multiagent chemotherapy regimens associated with better survival compared with less-aggressive regimens in older adults with Hodgkin lymphoma?

**Findings:**

In this population-based cohort study of 2686 patients aged 65 years or older with Hodgkin lymphoma, variability in the magnitude of the association between treatment intensity and mortality by stage and cause-specific mortality was found, possibly reflecting competing risks of death. However, full chemotherapy regimens tended to have the lowest mortality from any cause.

**Meaning:**

These findings suggest that full, multiagent chemotherapy regimens may be associated with better survival in older adults who can tolerate them.

## Teaser

This cohort study examines the association between treatment intensity and cause-specific mortality among older adults with Hodgkin lymphoma.

## Introduction

Hodgkin lymphoma (HL) is an aggressive hematologic cancer with increased incidence during adolescence and young adulthood and a second increase during late adulthood (age 70-80 years).^1,2^ Although HL is highly curable in younger patients, with 5-year survival rates exceeding 85%,^1,2^ older patients have 5-year survival rates lower than 60%.^1,3^ Worse outcomes in older patients may reflect different biological factors, competing risks of death, treatment-related toxic effects, reluctance to treat older patients aggressively, and end-of-life preferences.^4,5,6,7,8,9^ Current first-line treatment for HL, particularly in younger patients, includes multiagent chemotherapy, such as ABVD (adriamycin or doxorubicin, bleomycin, vinblastine, and dacarbazine) or BEACOPPesc (bleomycin, etoposide, adriamycin or doxorubicin, cyclophosphamide, oncovin or vincristine, procarbazine, and prednisone-escalated), with or without radiotherapy (RT).^10^ The number of chemotherapy cycles varies by disease stage, early response to treatment, and patients’ ability to tolerate treatment. In addition, older adults, particularly those with comorbidities, may not tolerate multiagent chemotherapy regimens and may be treated with partial chemotherapy regimens or palliative approaches.^4,10,11,12^

Although survival in older patients with HL may not achieve the levels of younger patients, opportunities exist to select treatments that optimize survival. However, studying the association between treatment intensity and survival in older adults is challenging because they are typically excluded from clinical trials.^13^ National registries, such as Surveillance, Epidemiology and End Results (SEER)–Medicare, provide real-world data as an alternative to clinical trials. Therefore, we examined the association between treatment intensity and cause-specific mortality among older adults with HL using SEER-Medicare data. We hypothesized that in unadjusted analyses, treatment with full chemotherapy regimens would have better survival, whereas those receiving no treatment would have worse survival. We hypothesized that adjustment for confounding would attenuate these associations.

## Methods

### Sample

This cohort study used SEER-Medicare data from 1999 to 2016. Patients were aged 65 years or older at diagnosis with incident classic HL, as defined by SEER registry histological data. Patients had to be eligible for at least 3 years of follow-up in the SEER registry to capture 3-year survival data. The cohort was restricted to patients with Medicare Part A and B fee-for-service coverage for 6 months before and 1 year after diagnosis (or until date of death) to fully capture treatment claims, thereby restricting diagnoses to 2000 to 2013. Exclusion criteria were missing diagnosis month, unknown diagnostic confirmation, diagnosis reported only from autopsy or death certificate, another cancer diagnosis less than 6 months before HL diagnosis, no claims within 6 months of diagnosis, and unknown stage.^12^ Patients with only 1 to 10 claims within 6 months of diagnosis were required to have 1 or more HL-related claim.

This study followed the Strengthening the Reporting of Observational Studies in Epidemiology (STROBE) reporting guideline.^14^ The institutional review board at Tufts Medical Center deemed this study exempt from review because data were publicly available and deidentified. Therefore, consent was not required in accordance with 45 CFR §46.

### Three-Year Survival

We used month and year of HL diagnosis and death from SEER registry data. Overall survival included all causes of death, whereas cause-specific mortality was classified into death from HL or all other causes of death based on SEER categorization. Time from diagnosis to death (in months) was calculated, with censoring at 3 years after diagnosis.

### First-line Treatment

First-line treatment initiated within 4 months of diagnosis was determined from inpatient, outpatient, and physician or supplier claims using chemotherapy J codes, Healthcare Common Procedure Coding System codes, and Diagnosis Related Group codes and was categorized as (1) full chemotherapy regimens (ie, full regimen), (2) partial chemotherapy regimen (ie, partial regimen), (3) single chemotherapy agent or RT (referred to hereafter as single agent or RT), or (4) no treatment. Full regimens were based on National Comprehensive Cancer Network guidelines and established chemotherapy regimens used during the study window, with a focus on older patients.^10,12,15,16,17,18,19^ Although the number of recommended chemotherapy cycles varied by disease stage, we categorized treatment on the basis of the first 2 cycles, which corresponded to the fewest number of cycles recommended for early stage HL and the fewest number of cycles before response-based treatment adaptation.^10^ In addition, using 2 cycles reduces immortal time bias and time-varying confounding (see the Statistical Analysis subsection). To be classified as receiving a full regimen, patients had to receive all drugs for 2 cycles, except orally administered drugs (eg, steroids and procarbazine), which were available only as Medicare Part D pharmacy claims for part of the study period. In light of research showing that bleomycin should be used cautiously in older adults,^10,20^ ABVD both with and without bleomycin was considered a full regimen. Partial regimens included any multiagent chemotherapy regimen that did not meet full regimen criteria (eg, doxorubicin and vinblastine alone). Single agent or RT included patients treated with 1 chemotherapy agent at a time or RT. No treatment was defined by no claims for these treatments within 4 months of diagnosis.

### Covariates

Potential confounders of the association between treatment and mortality included patient, disease, and geographical characteristics. Diagnosis date, age at diagnosis, gender, race or ethnicity (extracted from the SEER registry, which uses multiple data sources), marital status, HL histology, Ann Arbor stage, and B symptoms were defined from SEER registry data. Race and ethnicity were assessed in this study because disparities in cancer treatment and outcomes by race and ethnicity have been observed elsewhere.^12,21,22^ Medicaid dual eligibility was defined using the state buy-in indicator. Frailty and comorbidity in the 6 months before HL diagnosis were defined using claims-based algorithms.^23,24^ Frailty, a probability score, was converted to 1 to 100, where higher scores indicate higher probability of frailty. The current HL cancer was excluded from the comorbidity index. Using the comorbidity index, a separate cardiac comorbidity indicator was created for myocardial infarction or congestive heart failure, according to their association with treatment.^25^ Prior cancer was defined as having a SEER-Medicare entry for any cancer more than 6 months before HL diagnosis.

Geographical characteristics included region, population density, and presence of a hospital providing chemotherapy within the health service area. Region and population density were determined from the Medicare enrollment file. Population density was dichotomized as more populated (big metropolitan, metropolitan, and urban) and less populated (less urban and rural). Regions included Northeast, Midwest, South, and West based on SEER registry data. The 2017 to 2018 Area Health Resources Files Access System was used to determine whether there was a hospital providing chemotherapy in the health service area during the year of HL diagnosis.^26^

### Statistical Analysis

Analyses were done separately for early-stage (I and II) and advanced-stage (III and IV) disease because of differences in treatment and outcomes. Patient, disease, and geographical characteristics were described. Cell counts less than 11 were suppressed to avoid reidentification of patients.^27^ Analyses were conducted in R statistical software version 4.0.3 and RStudio statistical software version 1.4.1103 (both from R Project for Statistical Computing). Two-sided α was set to .05.

To address missing data in stage (160 patients), marital status (116 patients), race (12 patients), and B symptoms (665 patients), we created 10 multiply imputed data sets using the mice (multivariable imputation by chained equations) R package. Predictive mean matching with 10 iterations was done. Imputations were assessed for plausibility and convergence. The Rubin rule was used to pool results from multiply imputed data sets.^28^

To reduce the effect of immortal time bias, which could be caused by requiring patients to survive to the completion of all chemotherapy cycles to be classified as receiving full regimens, we conducted a landmark analysis.^29,30,31^ This shifted time 0 from diagnosis to 2 months after diagnosis, thereby removing patients who died within 2 months of diagnosis. We compared characteristics of those included and excluded in the landmark analysis to understand generalizability of our findings.

Kaplan-Meier plots of 3-year overall survival by treatment were estimated. Cox proportional hazards models with a competing risk framework were used to estimate hazard ratios (HRs) and 95% CIs for the association between first-line treatment and 3-year cause-specific hazard of mortality (HL specific or other-cause specific).^32^ Multivariable adjustment and propensity score weighting were used to adjust for confounding. The multivariable analysis adjusted for all covariates. The propensity score was fit using a generalized boosted model, which allowed for the 4 treatment categories (eAppendix in the Supplement).^33,34,35^ All disease-related variables were included in the propensity score model, as were variables that were related to both treatment and 3-year survival at *P* < .20. Balance between treatment groups was improved after propensity score weighting (eTable 1 in the Supplement). The propensity score–weighted Cox proportional hazards models also adjusted for all covariates.

An E-value was computed to quantify the minimum strength of association that an unmeasured confounder would need to have with both treatment and cause-specific mortality to completely explain away a significant association between treatment and cause-specific mortality based on the multivariable model.^36,37,38^ E-values were computed on the basis of whichever level of treatment had a HR that was closest to the null, but still significant, which provided a conservative estimate.

The proportional hazards assumption for the Cox models was assessed using correlations between Schoenfeld residuals and time, plots of Schoenfeld residuals over time, and interactions with step functions of time.^39^ Linearity of continuous covariates was assessed with plots of Martingale residuals. Data analysis was performed from April 2020 to June 2021.

## Results

### Sample

The cohort included 2686 patients (mean [SD] age, 75.7 [6.9] years; 1333 men [50%]), of whom 1307 (49%) had early-stage disease and 1379 (51%) had advanced-stage disease. Patient and geographical characteristics were similar across disease stage, but there were some differences in disease and treatment characteristics by disease stage (Table 1). Compared with the 2686 patients included in the analysis, 317 patients excluded from the landmark analysis were older (79.0 vs 75.7 years), had worse frailty (26.5 vs 15.3) and comorbidity (3 vs 1.8) scores, and had more advanced disease stage (stage IV disease, 140 patients [44%] vs 632 patients [24%]) (eTable 2 in the Supplement).

**Table 1.** Characteristics of Patients at Hodgkin Lymphoma Diagnosis by Stage

Characteristic	Patients, No. (%)		
	Overall (N = 2686)	Early stage (n = 1307)	Advanced stage (n = 1379)
Patient factors			
Age, mean (SD), y	75.7 (6.9)	75.8 (7.2)	75.5 (6.7)
Age category, y			
65-69	626 (23)	305 (23)	321 (23)
70-74	654 (24)	310 (24)	344 (25)
75-79	614 (23)	280 (21)	334 (24)
≥80	792 (29)	412 (32)	380 (28)
Sex			
Male	1333 (50)	638 (49)	695 (50)
Female	1353 (50)	669 (51)	684 (50)
Race and ethnicity			
Black, non-Hispanic	129 (5)	69 (5)	60 (4)
Hispanic	219 (8)	97 (7)	122 (9)
Other race, non-Hispanic^a^	87 (3)	33 (3)	54 (4)
White, non-Hispanic	2251 (84)	1108 (85)	1143 (83)
Marital status			
Married	1588 (59)	763 (58)	826 (60)
Single, divorced, or widowed	1098 (41)	544 (42)	554 (40)
Medicaid dual enrolled	346 (13)	159 (12)	187 (14)
Frailty score, mean (SD)	15.3 (12.4)	15.0 (12.4)	15.6 (12.3)
Comorbidity index, mean (SD)	1.8 (1.6)	1.7 (1.6)	2.0 (1.7)
Any comorbidity	2075 (77)	977 (75)	1098 (80)
Cardiac comorbidity	620 (23)	280 (21)	340 (25)
Prior cancer	426 (16)	210 (16)	216 (16)
Disease and treatment factors			
Diagnosis year			
2000-2004	860 (32)	468 (36)	392 (28)
2005-2009	1085 (40)	539 (41)	546 (40)
2010-2013	741 (28)	300 (23)	441 (32)
Histology			
Nodular sclerosis	1015 (38)	524 (40)	491 (36)
Mixed cellularity	571 (21)	288 (22)	283 (21)
Lymphocyte rich	118 (4)	83 (6)	35 (3)
Lymphocyte depleted	72 (3)	31 (2)	41 (3)
Not otherwise specified	910 (34)	381 (29)	529 (38)
Stage^b^			
I	659 (25)	643 (49)	0
II	659 (25)	664 (51)	0
III	736 (27)	0	756 (55)
IV	632 (24)	0	623 (45)
B symptoms	1193 (44)	426 (33)	767 (56)
First-line treatment			
Full regimen	1314 (49)	568 (43)	746 (54)
Partial regimen	469 (17)	235 (18)	234 (17)
Single agent or radiotherapy	383 (14)	259 (20)	124 (9)
None^c^	520 (19)	245 (19)	275 (20)
Geographical factors			
Region			
Northeast	639 (24)	321 (25)	318 (23)
Midwest	356 (13)	174 (13)	182 (13)
South	633 (24)	328 (25)	305 (22)
West	1058 (39)	484 (37)	574 (42)
Urban vs rural			
More populated	2382 (89)	1143 (87)	1239 (90)
Less populated	304 (11)	164 (13)	40 (10)
Hospital with chemotherapy in health service area	2572 (96)	1249 (96)	1323 (96)

^a^ Other includes Asian, Pacific Islander, multiracial, or any other race.

^b^ Overall and stage-specific frequencies differ because of the multiple imputation process.

^c^ No treatment refers to no claims for chemotherapy or radiotherapy.

### Association Between Treatment and Cause-Specific Mortality in Early-Stage Disease

By 3 years, 228 patients had died from HL, 281 from other causes, and 798 were still alive. For both causes of death, results from multivariable and propensity score–weighted models had attenuated HRs compared with unadjusted models (Figure panel A and Table 2); results from the multivariable model are described. For HL-specific mortality, HRs were higher for partial regimens (HR, 1.77; 95% CI, 1.22-2.57) or no treatment (HR, 1.91; 95% CI, 1.31-2.79) compared with full regimens; there was no difference between single agent or RT (HR, 1.37; 95% CI, 0.92-2.06) and full regimens. For other-cause mortality, HRs were higher for partial regimens (HR, 1.69; 95% CI, 1.18-2.44), single agent or RT (HR, 1.62; 95% CI, 1.13-2.33), or no treatment (HR, 2.71; 95% CI, 1.95-3.78) compared with full regimens.

**Figure.**
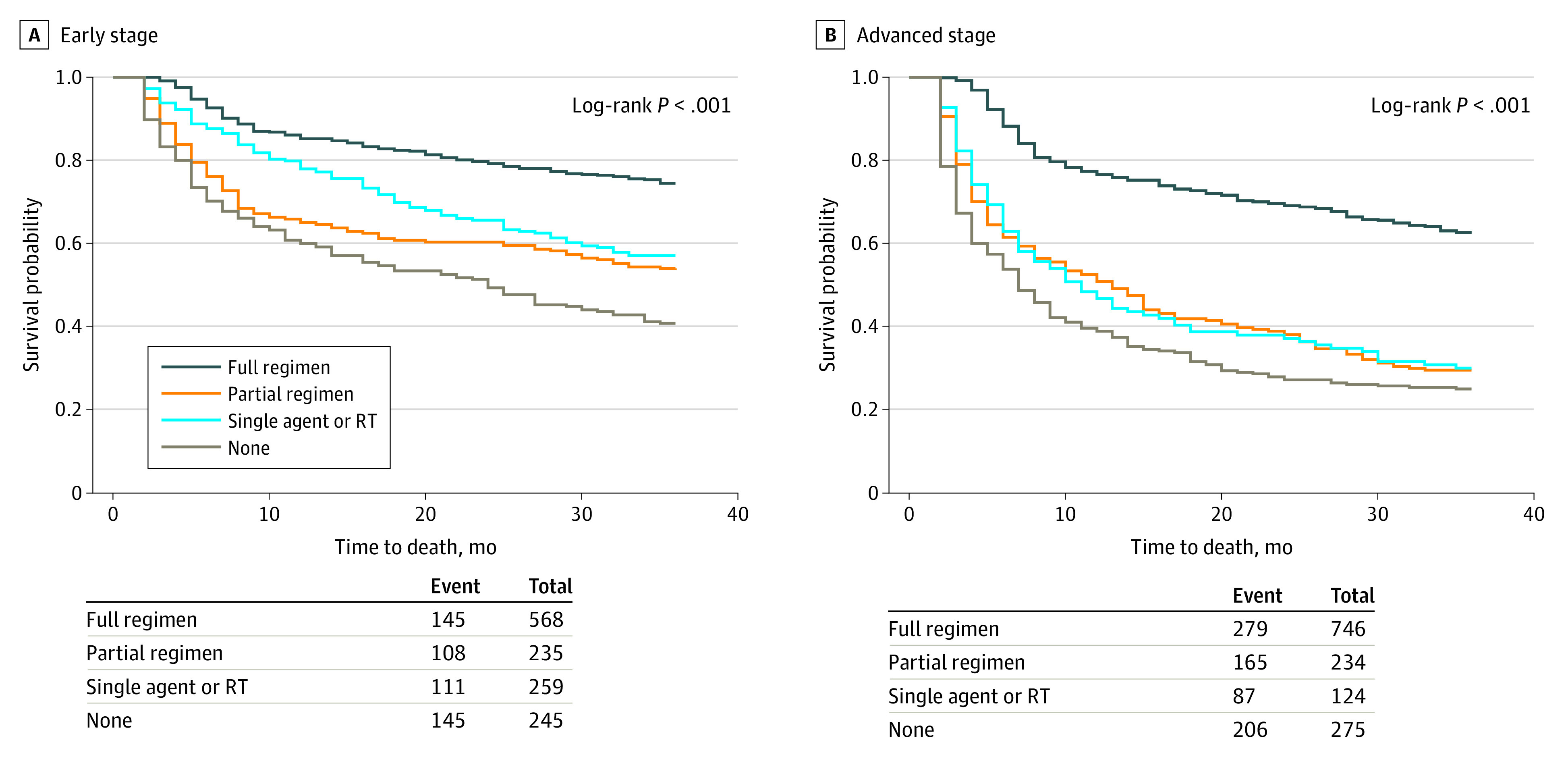
Kaplan-Meier Plots for 3-Year Overall Survival by First-line Treatment Data are shown for patients with early-stage disease (A) and advanced-stage disease (B). Numbers of patients at risk are not shown to maintain patient confidentiality. RT indicates radiotherapy.

**Table 2.** Unadjusted, Multivariable, and Propensity Score–Weighted Results From Cox Proportional Hazards Models of 3-Year Cause-Specific Mortality for 1307 Patients With Early-Stage Disease

Variable	HR (95% CI)					
	Hodgkin lymphoma–specific mortality (n = 228)^a^			Other causes of mortality (n = 281)^a^		
	Unadjusted	Multivariable^b^	Propensity score weighted^b^	Unadjusted	Multivariable^b^	Propensity score weighted^b^
First-line treatment						
Full regimen	1 [Reference]	1 [Reference]	1 [Reference]	1 [Reference]	1 [Reference]	1 [Reference]
Partial regimen	2.15 (1.51-3.07)	1.77 (1.22-2.57)	1.59 (1.12-2.25)	2.27 (1.60-3.22)	1.69 (1.18-2.44)	1.91 (1.39-2.62)
Single agent or radiotherapy	1.53 (1.05-2.21)	1.37 (0.92-2.06)	1.85 (1.32-2.59)	2.19 (1.57-3.06)	1.62 (1.13-2.33)	1.16 (0.81-1.65)
None	2.41 (1.70-3.41)	1.91 (1.31-2.79)	2.13 (1.52-2.99)	3.88 (2.84-5.30)	2.71 (1.95-3.78)	2.32 (1.69-3.18)
Patient factors						
Age, y	1.06 (1.04-1.08)	1.03 (1.00-1.06)	1.01 (0.98-1.04)	1.07 (1.06-1.09)	1.05 (1.02-1.08)	1.05 (1.02-1.08)
Male	1.07 (0.82-1.38)	1.31 (0.97-1.77)	1.67 (1.28-2.19)	1.31 (1.04-1.66)	1.56 (1.19-2.05)	1.52 (1.17-1.98)
Race and ethnicity						
Black, non-Hispanic	1.20 (0.70-2.07)	0.99 (0.55-1.78)	0.98 (0.62-1.55)	1.02 (0.60-1.71)	0.77 (0.44-1.35)	0.71 (0.41-1.22)
Hispanic	1.00 (0.61-1.64)	0.91 (0.52-1.61)	0.55 (0.32-0.96)	0.83 (0.51-1.34)	0.71 (0.41-1.21)	1.12 (0.72-1.75)
Other race, non-Hispanic^c^	0.80 (0.33-1.94)	0.79 (0.31-2.00)	0.59 (0.22-1.59)	0.49 (0.18-1.31)	0.55 (0.20-1.53)	0.54 (0.18-1.66)
White, non-Hispanic	1 [Reference]	1 [Reference]	1 [Reference]	1 [Reference]	1 [Reference]	1 [Reference]
Marital status						
Married	0.69 (0.53-0.90)	0.86 (0.63-1.17)	0.72 (0.55-0.94)	0.64 (0.50-0.81)	0.75 (0.57-0.99)	0.73 (0.56-0.94)
Single, divorced, or widowed	1 [Reference]	1 [Reference]	1 [Reference]	1 [Reference]	1 [Reference]	1 [Reference]
Medicaid dual enrolled	1.25 (0.86-1.81)	1.11 (0.72-1.73)	0.77 (0.49-1.20)	1.40 (1.01-1.94)	1.51 (1.03-2.23)	1.05 (0.71-1.55)
Frailty	1.04 (1.03-1.04)	1.02 (1.00-1.04)	1.03 (1.01-1.05)	1.04 (1.03-1.05)	1.01 (0.99-1.03)	1.01 (0.99-1.03)
Comorbidity index	1.16 (1.08-1.25)	1.08 (0.97-1.21)	1.07 (0.95-1.20)	1.22 (1.14-1.30)	1.09 (0.99-1.20)	1.11 (1.00-1.23)
Cardiac comorbidity	1.31 (0.97-1.77)	0.76 (0.52-1.12)	0.77 (0.51-1.14)	1.90 (1.48-2.45)	1.14 (0.82-1.58)	1.14 (0.80-1.63)
Prior cancer	0.89 (0.61-1.28)	0.96 (0.65-1.41)	0.85 (0.60-1.18)	1.35 (1.01-1.81)	1.32 (0.97-1.80)	1.05 (0.77-1.42)
Disease factors						
Diagnosis year	0.96 (0.93-1.00)	0.98 (0.94-1.02)	0.96 (0.93-1.00)	0.99 (0.96-1.02)	0.99 (0.95-1.02)	0.97 (0.93-1.00)
Histology						
Nodular sclerosis	1 [Reference]	1 [Reference]	1 [Reference]	1 [Reference]	1 [Reference]	1 [Reference]
Mixed cellularity	1.07 (0.77-1.49)	1.00 (0.71-1.40)	1.12 (0.84-1.49)	1.05 (0.76-1.45)	0.91 (0.65-1.27)	0.91 (0.66-1.25)
Lymphocyte rich	0.41 (0.19-0.88)	0.50 (0.23-1.10)	0.38 (0.16-0.89)	0.64 (0.35-1.17)	0.71 (0.38-1.30)	1.07 (0.63-1.81)
Lymphocyte depleted	3.33 (1.90-5.83)	2.18 (1.20-3.94)	1.17 (0.56-2.44)	1.41 (0.62-3.22)	0.94 (0.40-2.20)	0.85 (0.35-2.06)
Not otherwise specified	0.82 (0.59-1.14)	0.69 (0.49-0.98)	0.54 (0.38-0.75)	1.44 (1.10-1.89)	1.05 (0.78-1.40)	0.97 (0.73-1.29)
Stage						
I	0.74 (0.57-0.97)	0.74 (0.56-0.97)	0.82 (0.64-1.05)	1.04 (0.82-1.33)	0.93 (0.73-1.18)	0.84 (0.66-1.08)
II	1 [Reference]	1 [Reference]	1 [Reference]	1 [Reference]	1 [Reference]	1 [Reference]
B symptoms	2.16 (1.60-2.92)	1.94 (1.39-2.7)	2.31 (1.68-3.19)	1.58 (1.22-2.05)	1.48 (1.14-1.93)	1.41 (1.07-1.87)
Geographical factors						
Region						
Northeast	0.93 (0.65-1.32)	0.93 (0.64-1.35)	0.72 (0.51-1.00)	1.28 (0.94-1.74)	1.14 (0.83-1.58)	1.16 (0.85-1.57)
Midwest	1.09 (0.72-1.64)	1.12 (0.72-1.74)	0.95 (0.64-1.42)	0.83 (0.54-1.27)	0.82 (0.52-1.29)	0.85 (0.55-1.30)
South	1.26 (0.91-1.75)	1.21 (0.84-1.74)	1.06 (0.78-1.45)	1.58 (1.18-2.11)	1.59 (1.14-2.21)	1.34 (0.98-1.83)
West	1 [Reference]	1 [Reference]	1 [Reference]	1 [Reference]	1 [Reference]	1 [Reference]
Urban vs rural						
More populated	1 [Reference]	1 [Reference]	1 [Reference]	1 [Reference]	1 [Reference]	1 [Reference]
Less populated	1.15 (0.79-1.68)	1.02 (0.67-1.56)	0.57 (0.37-0.89)	1.11 (0.79-1.57)	1.00 (0.68-1.48)	1.48 (1.06-2.07)
Hospital with chemotherapy in health service area	1.00 (0.53-1.89)	0.80 (0.41-1.56)	1.26 (0.62-2.54)	0.88 (0.51-1.50)	0.70 (0.39-1.23)	1.11 (0.64-1.93)

Abbreviation: HR, hazard ratio.

^a^ At 3 years, 798 patients were alive.

^b^ Adjusted for all covariates: age, race and ethnicity, marital status, Medicaid dual enrollment, frailty score, comorbidity index, any cardiac comorbidity, prior cancer, year of diagnosis, histology, stage, B symptoms, census region, population density, and hospital with chemotherapy in health service area.

^c^ Other includes Asian, Pacific Islander, multiracial, or any other race.

### Association Between Treatment and Cause-Specific Mortality in Advanced-Stage Disease

By 3 years, 357 patients had died from HL, 380 from other causes, and 642 were still alive. For HL-specific mortality, the multivariable model was attenuated from unadjusted models, whereas the propensity score–weighted model was similar to unadjusted models. For other-cause mortality, results from multivariable and propensity score–weighted models were similar, both with attenuated HRs compared with unadjusted models (Figure panel B and Table 3). Results from the multivariable model are described. For HL-specific mortality, HRs were higher for partial regimens (HR, 3.26; 95% CI, 2.44-4.35), single agent or RT (HR, 2.85; 95% CI, 1.98-4.11), or no treatment (HR, 4.06; 95% CI, 3.06-5.37) compared with full regimens. For other-cause mortality, HRs were higher for partial regimens (HR, 1.76; 95% CI, 1.32-2.33), single agent or RT (HR, 1.65; 95% CI, 1.15-2.37), or no treatment (HR, 2.24; 95% CI, 1.71-2.94) compared with full regimens. Unexpectedly, lower other-cause mortality was found for non-Hispanic Black patients compared with non-Hispanic White patients (HR, 0.46; 95% CI, 0.25-0.82).

**Table 3.** Unadjusted, Multivariable, and Propensity Score Weighted Results From Cox Proportional Hazards Models of 3-Year Cause-Specific Mortality for 1379 Patients With Advanced-Stage Disease

Variable	HR (95% CI)					
	Hodgkin lymphoma–specific mortality (n = 357)^a^			Other causes of mortality (n = 380)^a^		
	Unadjusted	Multivariable^b^	Propensity score weighted^b^	Unadjusted	Multivariable^b^	Propensity score weighted^b^
First-line treatment						
Full regimen	1 [Reference]	1 [Reference]	1 [Reference]	1 [Reference]	1 [Reference]	1 [Reference]
Partial regimen	3.56 (2.69-4.70)	3.26 (2.44-4.35)	3.81 (3.03-4.79)	2.11 (1.61-2.77)	1.76 (1.32-2.33)	1.79 (1.45-2.22)
Single agent or radiotherapy	3.44 (2.44-4.85)	2.85 (1.98-4.11)	3.65 (2.85-4.66)	2.14 (1.52-3.01)	1.65 (1.15-2.37)	1.58 (1.24-2.01)
None	4.42 (3.40-5.76)	4.06 (3.06-5.37)	3.93 (3.11-4.97)	2.70 (2.10-3.48)	2.24 (1.71-2.94)	2.21 (1.78-2.74)
Patient factors						
Age, y	1.06 (1.04-1.07)	1.04 (1.02-1.07)	1.04 (1.02-1.06)	1.04 (1.03-1.06)	1.02 (0.99-1.04)	1.02 (1.00-1.04)
Male	1.05 (0.85-1.29)	1.14 (0.90-1.43)	1.11 (0.95-1.31)	0.99 (0.81-1.21)	1.17 (0.93-1.46)	1.41 (1.19-1.67)
Race and ethnicity						
Black, non-Hispanic	0.85 (0.50-1.46)	0.73 (0.41-1.29)	1.11 (0.77-1.59)	0.73 (0.42-1.28)	0.46 (0.25-0.82)	0.56 (0.37-0.87)
Hispanic	1.11 (0.78-1.57)	1.22 (0.81-1.84)	1.03 (0.78-1.37)	1.00 (0.7-1.43)	0.98 (0.65-1.48)	0.94 (0.70-1.27)
Other race, non-Hispanic^c^	0.89 (0.5-1.59)	0.93 (0.51-1.72)	1.04 (0.71-1.53)	1.11 (0.67-1.84)	1.20 (0.70-2.06)	0.73 (0.46-1.15)
White, non-Hispanic	1 [Reference]	1 [Reference]	1 [Reference]	1 [Reference]	1 [Reference]	1 [Reference]
Marital status						
Married	0.85 (0.69-1.05)	1.04 (0.82-1.33)	0.94 (0.79-1.12)	0.67 (0.55-0.82)	0.80 (0.63-1.01)	0.77 (0.65-0.91)
Single, divorced, or widowed	1 [Reference]	1 [Reference]	1 [Reference]	1 [Reference]	1 [Reference]	1 [Reference]
Medicaid dual enrolled	1.09 (0.81-1.48)	0.94 (0.66-1.34)	1.05 (0.83-1.34)	1.44 (1.10-1.88)	1.34 (0.98-1.83)	1.36 (1.08-1.71)
Frailty	1.03 (1.02-1.03)	1.00 (0.98-1.01)	0.99 (0.98-1.01)	1.03 (1.02-1.04)	1.01 (1.00-1.02)	1.01 (1.00-1.02)
Comorbidity index	1.16 (1.09-1.22)	1.09 (1.02-1.18)	1.14 (1.08-1.21)	1.20 (1.14-1.27)	1.09 (1.01-1.17)	1.11 (1.05-1.18)
Cardiac comorbidity	1.60 (1.27-2.01)	1.08 (0.81-1.44)	1.16 (0.94-1.45)	1.91 (1.54-2.37)	1.30 (0.99-1.69)	1.18 (0.95-1.47)
Prior cancer	0.96 (0.72-1.27)	0.92 (0.69-1.25)	1.03 (0.84-1.26)	1.14 (0.88-1.49)	1.23 (0.94-1.62)	1.13 (0.92-1.38)
Disease factors						
Diagnosis year	0.96 (0.94-0.99)	0.98 (0.95-1.01)	0.98 (0.96-1.00)	0.98 (0.95-1.01)	0.99 (0.96-1.01)	1.00 (0.98-1.02)
Histology						
Nodular sclerosis	1 [Reference]	1 [Reference]	1 [Reference]	1 [Reference]	1 [Reference]	1 [Reference]
Mixed cellularity	0.97 (0.72-1.31)	0.94 (0.69-1.28)	1.05 (0.85-1.29)	1.24 (0.94-1.64)	1.18 (0.89-1.57)	1.03 (0.83-1.28)
Lymphocyte rich	0.62 (0.27-1.40)	0.74 (0.32-1.72)	1.79 (1.16-2.78)	0.70 (0.33-1.50)	0.79 (0.36-1.71)	0.43 (0.21-0.91)
Lymphocyte depleted	1.73 (1.01-2.96)	1.67 (0.97-2.90)	1.80 (1.19-2.73)	1.66 (0.95-2.88)	1.47 (0.84-2.59)	1.32 (0.83-2.10)
Not otherwise specified	1.27 (1.0-1.61)	1.15 (0.90-1.47)	1.03 (0.85-1.23)	1.31 (1.03-1.66)	1.19 (0.93-1.51)	1.01 (0.84-1.21)
Stage						
III	0.69 (0.55-0.85)	0.71 (0.57-0.88)	0.64 (0.55-0.75)	0.83 (0.67-1.02)	0.87 (0.71-1.08)	0.82 (0.7-0.97)
IV	1 [Reference]	1 [Reference]	1 [Reference]	1 [Reference]	1 [Reference]	1 [Reference]
B symptoms	1.43 (1.11-1.82)	1.62 (1.27-2.07)	2.57 (2.15-3.07)	1.28 (1.02-1.60)	1.34 (1.06-1.70)	1.4 (1.14-1.74)
Geographical factors						
Region						
Northeast	0.95 (0.72-1.25)	0.91 (0.68-1.22)	0.95 (0.77-1.17)	1.09 (0.84-1.42)	1.13 (0.86-1.50)	1.09 (0.88-1.35)
Midwest	0.85 (0.60-1.20)	0.92 (0.64-1.33)	0.92 (0.71-1.18)	0.95 (0.68-1.32)	1.03 (0.73-1.47)	1.10 (0.85-1.42)
South	1.24 (0.96-1.61)	1.12 (0.83-1.51)	1.02 (0.82-1.26)	1.36 (1.05-1.76)	1.35 (1.01-1.81)	1.39 (1.12-1.74)
West	1 [Reference]	1 [Reference]	1 [Reference]	1 [Reference]	1 [Reference]	1 [Reference]
Urban vs rural						
More populated	1 [Reference]	1 [Reference]	1 [Reference]	1 [Reference]	1 [Reference]	1 [Reference]
Less populated	1.23 (0.89-1.70)	1.16 (0.81-1.66)	1.22 (0.97-1.55)	1.22 (0.89-1.67)	1.10 (0.77-1.57)	0.89 (0.69-1.16)
Hospital with chemotherapy in health service area	1.03 (0.59-1.80)	1.17 (0.65-2.08)	1.20 (0.78-1.84)	0.59 (0.39-0.91)	0.62 (0.40-0.97)	0.67 (0.47-0.95)

Abbreviation: HR, hazard ratio.

^a^ At 3 years, 642 patients were alive.

^b^ Adjusted for all covariates: age, race and ethnicity, marital status, Medicaid dual enrollment, frailty score, comorbidity index, any cardiac comorbidity, prior cancer, year of diagnosis, histology, stage, B symptoms, census region, population density, and hospital with chemotherapy in health service area.

^c^ Other includes Asian, Pacific Islander, multiracial, or any other race.

### E-value Analysis

 The observed HR of 1.62 for single agent or RT and other-cause mortality among patients with early-stage disease could be explained by an unmeasured confounder that was associated with both treatment and other-cause mortality with a HR of 2.14, above and beyond measured confounders. See the eFigure in the Supplement for additional data.

## Discussion

In this large, population-based cohort study of older patients with HL, we found a significant association between treatment intensity and 3-year cause-specific mortality, even after adjustment for confounders such as age, comorbidity, and frailty. Patients with early-stage and advanced-stage disease generally had lower HL-specific and other-cause mortality when treated with full chemotherapy regimens than partial regimens, single agent or RT, or no treatment.

For early-stage disease, HRs for single agent or RT and no treatment (compared with full regimens) were higher for other-cause mortality than HL-specific mortality. These differences could be reflecting competing causes of death; furthermore, less-aggressive treatment may have been selected because of competing risks, which may not have been fully accounted for in our models. For advanced-stage disease, HRs for partial regimens, single agent or RT, and no treatment (compared with full regimens) were higher for HL-specific mortality than other-cause mortality. This suggests that some older patients could minimize their mortality, especially HL-specific mortality, with more-intensive chemotherapy if they are able to tolerate it. Interestingly, the HRs for less-intensive chemotherapy for advanced-stage disease were larger than those for early-stage disease, suggesting that treatment intensity matters more for advanced-stage disease. Of note, the magnitude of our HRs is similar to those observed in another study^40^ comparing different treatment intensities in older patients with HL (eg, reduced-intensity VEPEMB [vinblastine, cyclophosphamide or endoxan, procarbazine, etoposide, mitoxantrone, and bleomycin] vs ABVD had an HR for progression-free survival of 2.19). Although treatment-related toxic effects were not assessable in this study, they likely are associated with both treatment selection and mortality.

Aside from treatment, other factors were associated with mortality, with some differences by stage and cause of death. B symptoms were associated with worse HL-specific and other-cause mortality in early-stage and advanced-stage models. Prior research shows that B symptoms are common in older patients^11^ and are associated with worse outcomes.^21^ Higher stage disease was associated with worse HL-specific mortality in both early-stage and advanced-stage disease. Interestingly, older age was associated with worse other-cause mortality only in early-stage disease, whereas older age was associated with worse HL-specific mortality in advanced-stage disease. One explanation is that older patients with early-stage disease are dying from other causes, whereas older patients with advanced-stage disease are dying from HL. For advanced-stage disease, more comorbidities were associated with worse HL-specific and other-cause mortality. Our results support prior research^41^ that found a high prevalence of comorbidities in older patients with HL and worse outcomes in patients with comorbidities. Differences in mortality by Medicaid dual eligibility and region may reflect disparities in care or outcomes,^42,43,44^ whereas differences by gender and histology support previous research in HL.^21,45^ Lower other-cause mortality for Black non-Hispanic patients with advanced disease was unexpected, but may be associated with adjustment for factors contributing to racial and ethnic disparities.^46^

These findings have implications for clinical practice. Even after adjustment for confounders, patients receiving full regimens had the best survival. Therefore, full regimens could be considered for patients who can tolerate them and for whom full regimens align with treatment preferences. Age should not be the only deciding factor in treatment. Older patients without comorbidity or frailty may tolerate intense chemotherapy regimens. Integration of frailty and geriatric assessment into clinical care could inform treatment decisions.^6,47,48,49,50^ We observed that patients also experience competing risks of death from non-HL causes. Although not assessed here, treatment preferences likely are associated with treatment intensity and survival. For example, some patients may prefer length of life vs treatment-related toxic effects or quality of life, or vice versa, but data about preferences among older patients with HL are scarce.^51^ These findings highlight the importance of patient-physician discussion about treatment-related toxic effects, quality of life, and treatment goals.^47^ Finally, recently approved novel agents, such as immune checkpoint inhibitors, may offer alternatives to full chemotherapy regimens among older, frail patients.^6,10,52^

### Limitations

Limitations of our study design include confounding by indication and possible immortal time bias. We sought to address confounding with multivariable adjustment and propensity score weighting. Despite planned analysis, we were unable to conduct an instrumental variable analysis based on naturally occurring geographical variations in physician treatment preferences because the available geographical regions were either too large (SEER registry) or too small (hospital referral region).^53,54,55^ We did observe attenuation of treatment effect sizes after multivariable adjustment, indicating successful adjustment for measured confounders. Improved balance across measured confounders after propensity score weighting also indicates reduced confounding. Although unmeasured confounders (eg, bulky disease or performance status) may explain the remaining association, the E-value analysis found that any unmeasured confounder would need to have a stronger association with treatment and survival than most measured confounders. On the basis of the International Prognostic Score in HL, the strongest factor associated with risk of disease progression (low serum albumin) had a risk ratio of 1.49,^45^ indicating that although confounding could explain this association, it is unlikely. We addressed immortal time bias using a landmark analysis, which required patients to survive at least 2 months from diagnosis. Although this excluded older and sicker patients, they were likely part of a different population for whom treatment was not considered; therefore, these results generalize to patients surviving at least 2 months.

We acknowledge this study’s other limitations. SEER-Medicare is the largest longitudinal population-based database of older adults with cancer in the US, but patients in the database are not necessarily representative of all older patients with HL.^56^ To determine first-line treatment using claims data, patients were limited to those with Medicare Part A and B fee-for-service, thus limiting generalizability by excluding patients with Medicare Advantage who tend to be healthier, low-to-middle income, and from more populated areas.^57^ Our treatment definition was based on the first 2 cycles of chemotherapy. Although this allowed us to capture the fewest number of recommended cycles and to reduce time-varying confounding and immortal time bias, the definition may not align with what clinicians and guidelines considered full chemotherapy regimens, especially for advanced disease.^10^ In addition, given that SEER-Medicare data do not include information on chemotherapy dosages or treatment-related toxic effects, we were unable to consider dose modifications and were unable to adjust for potential confounding by time-varying treatment-related toxic effects. No treatment was assumed on the basis of the lack of any treatment-related claims, which could result in treatment misclassification. However, the following evidence indicates this was not a major issue: all patients were required to have medical claims at the time of their HL diagnosis, patients not receiving treatment because they died within 2 months of diagnosis were excluded from the landmark analysis, and our prior work^12^ found an explanation for why most patients did not receive treatment (eg, hospice, delayed treatment, or death).

## Conclusions

We present a large population-based analysis examining treatment intensity and survival among older patients with newly diagnosed HL. We found variability in the magnitude of the association between treatment intensity and mortality by stage and cause-specific mortality, possibly reflecting competing risks of death. Full chemotherapy regimens may be associated with better survival in older adults who can tolerate them. Consideration of patient age, competing risks of death, and geriatric assessment (including both frailty and comorbidity), as well as discussion of treatment preferences, can better inform treatment selection and likely translate to improved outcomes.

## Notes

Supplement. **eAppendix.** Supplemental Methods **eTable 1.** Comparison of Key Factors by Treatment Group Based on Observed Data and Propensity Score Weighted Results **eTable 2.** Comparison of Key Factors by Inclusion in Landmark Analysis **eFigure.** E-Value Analysis
eReferences
