# Supplementary material for: Association of Treatment Intensity With Survival in Older Patients With Hodgkin Lymphoma
Source: JAMA Netw Open. 2021 Oct 21;4(10):e2128373. doi: 10.1001/jamanetworkopen.2021.28373 (PMC8531990; doi:10.1001/jamanetworkopen.2021.28373)
Supplement: Supplement. — eAppendix. Supplemental Methods eTable 1. Comparison of Key Factors by Treatment Group Based on Observed Data and Propensity Score Weighted Results eTable 2. Comparison of Key Factors by Inclusion in Landmark Analysis eFigure. E-Value Analysis eReferences [file jamanetwopen-e2128373-s001.pdf]

Supplemental Online Content

Rodday AM, Hahn T, Kumar AJ, et al. Association of treatment intensity with survival in older patients with Hodgkin lymphoma. *JAMA Netw Open*. 2021;4(10):e2128373. doi:10.1001/jamanetworkopen.2021.28373

eAppendix. Supplemental Methods
eTable 1. Comparison of Key Factors by Treatment Group Based on Observed Data and Propensity Score Weighted Results
eTable 2. Comparison of Key Factors by Inclusion in Landmark Analysis
eFigure. E-Value Analysis
eReferences

This supplemental material has been provided by the authors to give readers additional information about their work.

© 2021 Rodday AM et al. *JAMA Network Open.*

eAppendix. Supplemental Methods
Propensity score analysis

Propensity score analysis involves a two-part process where (1) a regression model is fit to predict the probability treatment (referred to as the propensity score) and (2) the propensity score is applied to the analysis of the relationship between the treatment and outcome using one of several techniques (e.g., matching, weighting).[1] Propensity scores are commonly used for two treatment options, but methods exist for using the propensity score with multiple treatment categories.[2,3] We used a generalized boosted model (using the mnps function in the R package twang) to estimate propensity scores, which then produced weights that could be incorporated into the Cox PH model. Generalized boosted models use an iterative process with multiple regression trees to capture the relationship between treatment and pre-treatment variables, allowing for non-linear relationships and without over-fitting the data. Stopping rules are used to select the trees that achieve the best balance across treatment groups based on user-defined criteria. We selected the criteria that achieved the best balance in covariates across treatment groups, maximized overlap in propensity scores across treatment, and resulted in the largest effective sample size. For the early stage model, the stopping rule based on summarizing covariates using the mean of the Kolmogorov-Smirnov (KS) balance metrics achieved the best balance across the treatment groups; for the advanced stage model, the stopping rule for the maximum of the KS balance metrics achieved the best balance across the treatment groups (eTable 1 in the Supplement). Patient, disease, and geographic characteristics included in the propensity score were described across treatment group while incorporating propensity score weights to assess balance (reported as means and standard errors [SE] for continuous variables or mean proportions and SE for binary/categorical variables). We chose to estimate the average treatment effect among the treated (ATT) in the population because we wanted to know the effect of receiving full chemotherapy regimens rather than the treatment the patient received. All disease-related variables were included in the propensity score model, as were variables that were related to both treatment and 3-year survival at $p<0.2$.

E-value analysis

E-values quantify the minimum strength of association that an unmeasured confounder would need to have with both the treatment and mortality to completely explain away a significant association between treatment and mortality. [4-6] We calculated the E-value using the hazard ratio (HR) for whichever level of treatment had a HR that was closest to the null, but still statistically significant. This would provide the most conservative estimate of the E-value. When calculating the E-value, we used HR (outcome prevalence >15%) as the outcome type. The lowest possible E-value is 1 (indicating no unmeasured confounding is required to explain away the effect) and a higher E-value indicates that the confounder associations must be stronger to explain away the effect. The E-value sets the association between the confounder and outcome and the confounder and exposure to be equal. If one association is smaller than the E-value, then the other relationship must be larger; this trade-off is represented as the curve on the plot.

Across both stages of disease and both causes of mortality, the HR that was closest the the null, but still statistically significant was 1.62 for single agent/radiotherapy (RT) and other cause-mortality among early stage patients. The E-value analysis found that this observed HR of 1.62 could be explained away by an unmeasured confounder that was associated with both treatment and mortality with a HR of 2.14, above and beyond the measured confounders; the 95% CI could be moved to include the null by an unmeasured confounder that was associated with both the treatment and the outcome with a HR of 1.40 (eFigure 1 in the Supplement).

To give context to the E-value, we plotted points for measured confounders that had significant relationships with either treatment or mortality. Since treatment is a 4-level variable, we selected the treatment level with the strongest relationship with the confounder (i.e., farthest from the null). Only one measured confounder was above the E-value threshold: lymphocyte depleted histology. All of the other measured confounders were below the E-value threshold. We plotted a hypothetical unmeasured confounder that could have eliminated the observed relationship between treatment and mortality. To give additional context, based on the International Prognostic Score in HL, the risk factor with the strongest relationship with disease progression had a risk ratio of 1.49 (low serum albumin), indicating that confounding could explain the relationship, but it is unlikely.[7]

eTable 1. Comparison of Key Factors by Treatment Group Based on Observed Data and Propensity Score Weighted Results [a]

	Observed				Propensity score weighted			
	Full	Partial	Single/RT	None	Full	Partial	Single/RT	None
**Early stage**	568	n=235	n=259	n=245	n=58	n=385	n=386	n=390
**Mean age in years (SD)**	73.5 (6.1)	75.9 (6.3)	79.2 (7.5)	77.7 (7.7)	73.5 (0.3)	73.7 (0.5)	74.6 (0.5)	74.0 (0.6)
**Married, n (%)**	367 (65%)	146 (62%)	144 (56%)	106 (43%)	369 (65%)	227 (59%)	255 (66%)	242 (62%)
**Medicaid dual enrolled, n (%)**	55 (10%)	33 (14%)	32 (12%)	39 (16%)	55 (10%)	43 (11%)	40 (10%)	52 (13%)
**Mean frailty (SD)**	11.2 (9.2)	14.5 (10.5)	18.7 (12.8)	20.3 (16.6)	11.2 (0.4)	10.5 (0.5)	11.6 (0.7)	11.1 (0.6)
**Mean comorbidity (SD)**	1.4 (1.4)	2 (1.6)	1.6 (1.5)	2.1 (1.7)	1.4 (0.1)	1.4 (0.1)	1.4 (0.1)	1.4 (0.1)
**Cardiac comorbidity, n (%)**	81 (14%)	33 (14%)	63 (24%)	74 (30%)	81 (14%)	55 (14%)	50 (13%)	52 (13%)
**Diagnosis year, n (%)**								
2000-2004	176 (31%)	88 (37%)	119 (46%)	85 (35%)	176 (31%)	120 (31%)	139 (36%)	105 (27%)
2005-2009	245 (43%)	102 (43%)	101 (39%)	91 (37%)	245 (43%)	160 (42%)	185 (48%)	178 (46%)
2010-2013	147 (26%)	45 (19%)	39 (15%)	69 (28%)	147 (26%)	104 (27%)	63 (16%)	107 (27%)
**Histology, n (%)**								
Nodular Sclerosis	261 (46%)	93 (40%)	101 (39%)	69 (28%)	261 (46%)	177 (46%)	203 (53%)	170 (44%)
Mixed Cellularity	118 (21%)	63 (27%)	50 (19%)	57 (23%)	118 (21%)	83 (22%)	73 (19%)	76 (19%)
Lymphocyte Rich	29 (5%)	**	34 (13%)	**	29 (5%)	**	25 (6%)	**
Lymphocyte Depleted	12 (2%)	**	0 (0%)	**	12 (2%)	**	0 (0%)	**
NOS	148 (26%)	57 (24%)	74 (29%)	102 (42%)	148 (26%)	96 (25%)	85 (22%)	117 (30%)
**Stage, n (%)**								
I	262 (46%)	105 (45%)	163 (63%)	127 (52%)	262 (46%)	162 (42%)	199 (52%)	173 (44%)
II	306 (54%)	130 (55%)	96 (37%)	118 (48%)	306 (54%)	222 (58%)	187 (48%)	217 (56%)
**B symptoms, n (%)**	186 (33%)	90 (38%)	60 (23%)	90 (37%)	187 (33%)	131 (34%)	97 (25%)	133 (34%)
**Region, n (%)**								
Northeast	130 (23%)	62 (26%)	56 (22%)	73 (30%)	130 (23%)	113 (29%)	78 (20%)	102 (26%)
Midwest	86 (15%)	18 (8%)	39 (15%)	31 (13%)	86 (15%)	32 (8%)	51 (13%)	45 (12%)
South	86 (15%)	77 (33%)	63 (24%)	59 (24%)	129 (23%)	94 (24%)	124 (32%)	82 (21%)
West	223 (39%)	78 (33%)	101 (39%)	82 (33%)	223 (39%)	146 (38%)	134 (35%)	161 (41%)
**Advanced Stage**	n=746	n=234	n=124	n=275	n=746	n=637	n=540	n=624
**Mean age in years (SD)**	73.8 (5.8)	75.6 (6.3)	79.7 (6.7)	77.9 (7.6)	73.8 (0.2)	73.9 (0.4)	74.6 (0.7)	73.9 (0.4)
**Married, n (%)**	495 (66%)	130 (56%)	65 (52%)	135 (49%)	375 (66%)	239 (62%)	243 (63%)	250 (64%)
**Mean frailty (SD)**	12.2 (9.3)	16.1 (10)	22.5 (13.9)	21.2 (16.4)	12.2 (0.3)	12.3 (0.5)	12.2 (0.9)	12.2 (0.5)
**Mean comorbidity (SD)**	1.7 (1.6)	2.1 (1.7)	2.3 (1.7)	2.2 (1.9)	1.7 (0.1)	1.7 (0.1)	1.8 (0.2)	1.8 (0.1)

© 2021 Rodday AM et al. *JAMA Network Open.*

**Cardiac comorbidity, n (%)**	136 (18%)	67 (29%)	46 (37%)	91 (33%)	136 (24%)	134 (35%)	108 (28%)	121 (31%)
**Diagnosis year, n (%)**								
2000-2004	178 (24%)	83 (35%)	42 (34%)	89 (32%)	178 (31%)	175 (45%)	133 (34%)	154 (39%)
2005-2009	317 (42%)	81 (35%)	42 (34%)	106 (39%)	317 (56%)	226 (59%)	227 (59%)	266 (68%)
2010-2013	251 (34%)	70 (30%)	40 (32%)	80 (29%)	251 (44%)	236 (61%)	180 (47%)	204 (52%)
**Histology, n (%)**								
Nodular Sclerosis	269 (36%)	86 (37%)	50 (40%)	86 (31%)	269 (47%)	248 (64%)	175 (45%)	224 (57%)
Mixed Cellularity	163 (22%)	52 (22%)	21 (17%)	47 (17%)	163 (29%)	134 (35%)	88 (23%)	116 (30%)
Lymphocyte Rich	22 (3%)	**	**	**	22 (4%)	**	**	24 (6%)
Lymphocyte Depleted	24 (3%)	**	**	**	24 (4%)	**	**	19 (5%)
NOS	268 (36%)	86 (37%)	49 (40%)	126 (46%)	268 (47%)	230 (60%)	251 (65%)	241 (62%)
**Stage, n (%)**								
III	415 (56%)	117 (50%)	64 (52%)	144 (52%)	415 (73%)	327 (85%)	264 (68%)	334 (86%)
IV	331 (44%)	117 (50%)	60 (48%)	131 (48%)	331 (58%)	309 (80%)	275 (71%)	290 (74%)
**B symptoms, n (%)**	434 (58%)	125 (54%)	61 (49%)	146 (53%)	329 (58%)	223 (58%)	201 (52%)	211 (54%)
**Region, n (%)**								
Northeast	173 (23%)	51 (22%)	32 (26%)	62 (23%)	173 (30%)	137 (36%)	122 (32%)	128 (33%)
Midwest	113 (15%)	21 (9%)	16 (13%)	32 (12%)	113 (20%)	70 (18%)	91 (24%)	69 (18%)
South	144 (19%)	63 (27%)	30 (24%)	68 (25%)	144 (25%)	145 (38%)	117 (30%)	130 (33%)
West	316 (42%)	99 (42%)	46 (37%)	113 (41%)	316 (56%)	285 (74%)	209 (54%)	298 (76%)

NOS=not otherwise specified; RT=radiotherapy
** Cell counts suppressed
[a] Restricted to variables included in propensity score (differs by stage).

© 2021 Rodday AM et al. *JAMA Network Open.*

eTable 2. Comparison of Key Factors by Inclusion in Landmark Analysis

	Included in landmark analysis, n=2686	Excluded from landmark analysis, n=317
Patient Factors		
Age in years, mean (SD)	75.7 (6.9)	79.0 (6.9)
Age categorical, n (%)		
65-69 years	626 (23%)	35 (11%)
70-74 years	654 (24%)	52 (16%)
75-79 years	614 (23%)	68 (21%)
80+ years	792 (29%)	162 (51%)
Gender, n (%)		
Male	1333 (50%)	166 (52%)
Female	1353 (50%)	151 (48%)
Race/ethnicity, n (%)		
White/non-Hispanic	2251 (84%)	249 (79%)
Black/non-Hispanic	129 (5%)	18 (6%)
Hispanic	219 (8%)	37 (12%)
Other race/non-Hispanic	87 (3%)	18 (6%)
Marital status, n (%)		
Married	1588 (59%)	153 (48%)
Single/Divorced/Widowed	1098 (41%)	164 (52%)
Medicaid dual enrolled, n (%)	346 (13%)	69 (22%)
Frailty, mean (SD)	15.3 (12.4)	26.5 (15.7)
Comorbidity index, mean (SD)	1.8 (1.6)	3 (1.8)
Any comorbidity, n (%)	2075 (77%)	295 (93%)
Cardiac comorbidity, n (%)	620 (23%)	158 (50%)
Prior cancer, n (%)	426 (16%)	53 (17%)
Disease & Treatment Factors		
Diagnosis year, n (%)		
2000-2004	860 (32%)	102 (32%)
2005-2009	1085 (40%)	125 (39%)
2010-2013	741 (28%)	90 (28%)
Histology, n (%)		
Nodular Sclerosis	1015 (38%)	86 (27%)
Mixed Cellularity	571 (21%)	57 (18%)
Lymphocyte Rich	118 (4%)	**
Lymphocyte Depleted	72 (3%)	**
NOS	910 (34%)	144 (45%)
Stage, n (%)		
I	659 (25%)	46 (15%)
II	659 (25%)	49 (16%)
III	736 (27%)	81 (25%)
IV	632 (24%)	140 (44%)
B symptoms, n (%)	1193 (44%)	207 (65%)
First-line treatment, n (%)		
Full regimen	1314 (49%)	0 (0%)
Partial regimen	469 (17%)	28 (9%)
Single agent/RT	383 (14%)	13 (4%)
None	520 (19%)	276 (87%)
Geographic Factors		
Region, n (%)		
Northeast	639 (24%)	71 (22%)
Midwest	356 (13%)	43 (14%)

© 2021 Rodday AM et al. *JAMA Network Open.*

South	633 (24%)	79 (25%)
West	1058 (39%)	124 (39%)
Urban/Rural, n (%)		
More populated	2382 (89%)	277 (87%)
Less populated	304 (11%)	40 (13%)
Hospital with chemotherapy in HSA, n (%)	2572 (96%)	295 (93%)

NOS=not otherwise specified; HSA=health service area.
** Cell counts suppressed
[a] No treatment refers to no claims for chemotherapy or RT

© 2021 Rodday AM et al. *JAMA Network Open.*

eFigure. E-Value Analysis

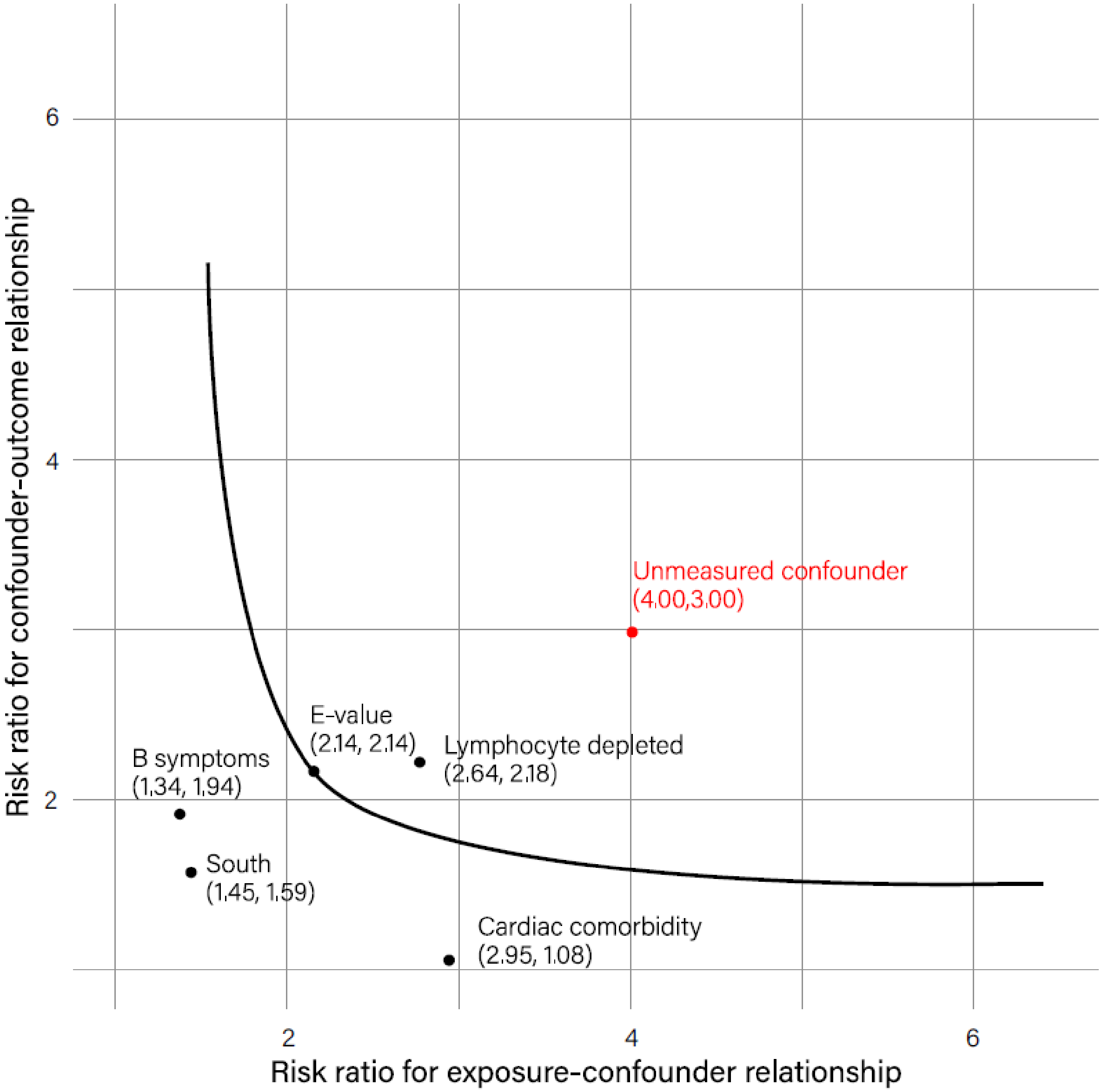

© 2021 Rodday AM et al. *JAMA Network Open.*

eReferences:

1. D'Agostino RB, Jr. Propensity score methods for bias reduction in the comparison of a treatment to a non-randomized control group. *Statistics in medicine.* Oct 15 1998;17(19):2265-2281.
2. McCaffrey DF, Griffin BA, Almirall D, Slaughter ME, Ramchand R, Burgette LF. A tutorial on propensity score estimation for multiple treatments using generalized boosted models. *Stat Med.* Aug 30 2013;32(19):3388-3414.
3. Propensity Scores for Multiple Treatments: A Tutorial for the MNPS Macro in the TWANG SAS Macros. RAND Corporation; 2015. https://www.rand.org/pubs/tools/TL169z1.html. Accessed 6/1/2020.
4. VanderWeele TJ, Ding P. Sensitivity Analysis in Observational Research: Introducing the E-Value. *Ann Intern Med.* Aug 15 2017;167(4):268-274.
5. Mathur MB, Ding P, Riddell CA, VanderWeele TJ. Web Site and R Package for Computing E-values. *Epidemiology.* Sep 2018;29(5):e45-e47.
6. Mathur M, Ding P, Riddell C, Simith L, VanderWeele T. E-Value Calculator. https://www.evalue-calculator.com/. Accessed 10/19/2020.
7. Hasenclever D, Diehl V. A prognostic score for advanced Hodgkin's disease. International Prognostic Factors Project on Advanced Hodgkin's Disease. *N Engl J Med.* Nov 19 1998;339(21):1506-1514.

© 2021 Rodday AM et al. *JAMA Network Open.*
